# Neuropilin-1 is associated with clinicopathology of gastric cancer and contributes to cell proliferation and migration as multifunctional co-receptors

**DOI:** 10.1186/s13046-016-0291-5

**Published:** 2016-01-22

**Authors:** Linhao Li, Xian Jiang, Qian Zhang, Xuesong Dong, Yuqiang Gao, Yuanlong He, Haiquan Qiao, Fangyu Xie, Xiangjun Xie, Xueying Sun

**Affiliations:** Department of General Surgery, Qingdao Municipal Hospital, Qingdao, 266011 China; Department of General Surgery, the First Affiliated Hospital of Harbin Medical University, No. 23 Youzheng Street, Harbin, 150001 China; Department of Obstetrics and Gynecology, Qingdao Municipal Hospital, Qingdao, 266011 China; Department of Gastroenterology, Qingdao Municipal Hospital, No. 1 Jiaozhou Road, Qingdao, 266011 China

**Keywords:** Neuropilin-1, Gastric cancer, Clinicopathology, Proliferation, Metastasis, Cellular signaling

## Abstract

**Background:**

Neuropilin-1 (NRP-1) is a transmembrane glycoprotein participating in the growth and metastasis of cancer cells as multifunctional co-receptors by interacting with the signaling pathways. However, its role in gastric cancer has not yet been clarified. This study aims to investigate whether NRP-1 expression is associated with the clinicopathology of gastric cancer, and involved in the growth and metastasis of gastric cancer cells.

**Methods:**

NRP-1 expression in clinical gastric cancer specimens was examined by immunohistochemistry and its association with clinicopathology analyzed. The expression of NRP-1 in a panel of human gastric cancer cells was examined by real-time RT-PCR and immunoblotting. Stable transfectants depleted of NRP-1, termed MGC-803-NRP^low^, were generated from MGC-803 cells. Cell proliferation was analyzed by the Cell Counting Kit-8 and Bromodeoxyuridine incorporation assays, and migrating ability analyzed by migration assays. The xenograft model was used to assess the effects of NRP-1 depletion on tumorigenesis, growth, metastasis and therapeutic potentials. The role of NRP-1 as co-receptors in the signaling pathways stimulated by ligands was examined. The key molecules involved in cell proliferation, migration and related signaling pathways were detected by immunoblotting.

**Results:**

Gastric cancer tissues expressed higher levels of NRP-1 compared to normal gastric mucosa. Its expression correlated with clinical staging, tumor differentiation and pathological types. NRP-1 depletion inhibited cell proliferation by inducing cell cycle arrest in the G1/S phase by upregulating p27, and downregulating cyclin E and cyclin-dependent kinase 2. NRP-1 depletion reduced the ability of cells to migrate by inhibiting the phosphorylation of focal adhesion kinase. NRP-1 depletion suppressed tumorigenesis, tumor growth and lung metastasis by inhibiting cell proliferation and tumor angiogenesis in situ. Therapeutic NRP-1 shRNA inhibited the growth of established BGC823 tumors. Depletion of NRP-1 inhibited the activation of VEGF/VEGFR2, EGF/EGFR and HGF/c-Met pathways stimulated by respective recombinant human VEGF-165, EGF and HGF proteins.

**Conclusions:**

The present results indicate that NRP-1 may be a potentially valuable biomarker and therapeutic target for gastric cancer.

**Electronic supplementary material:**

The online version of this article (doi:10.1186/s13046-016-0291-5) contains supplementary material, which is available to authorized users.

## Background

Neuropilin-1 (NRP-1), a transmembrane glycoprotein, was initially identified as a neuronal receptor for the class 3 semaphorins [1]. It is now known that NRP-1 also acts as multifunctional co-receptors participating in the initiation, growth and metastasis of cancer cells [1]. NRP-1 is overexpressed in several common types of cancer. More importantly, NRP-1 expression associates with the advanced stage of disease, and negatively correlates with the prognosis of hepatocellular carcinoma [2], colorectal cancer [3], and esophagus squamous cell carcinoma [4], indicating that NRP-1 is a potential molecular target for cancer therapy. Specific inhibition of NRP-1 has been shown to suppress the growth and metastasis of several types of cancer cells [5–9].

The mechanisms for the role of NRP-1 in cancer progression rely on its interactions with several key signaling pathways in cancer cells. NRP-1 interacts with vascular endothelial growth factor (VEGF) and its receptor VEGFR2, leading to the activation of this pathway and promoting tumor angiogenesis and growth [10–12]. NRP-1 also co-interacts with other important heparin-binding cytokines, such as epithelial growth factor (EGF) and its receptor (EGFR) [13], and hepatocyte growth factor (HGF) and its receptor c-Met [14], and activates these two pathways. The above three cellular signaling pathways are all shown to be involved in the progression of gastric cancer [15–18].

It has been reported that NRP-1 is expressed in human gastric cancer cells and in limited number of human gastric cancer tissues [15]. However, the role of NRP-1 in the progression of gastric cancer and the related mechanisms has not been fully elucidated. Gastric cancer is the third most common cause of cancer-related death in men worldwide [19]. Conventional adjuvant treatments have shown only modest effects on the survival of patients with advanced gastric cancer, and the development of molecular targeting drugs for gastric cancer is lagging behind some other cancers [20]. Therefore, it is essential for seeking novel molecular targets for combating gastric cancer. Here, we designed the present study aiming to investigate whether NRP-1 expression correlates with the clinicopathologic features of gastric cancer, and whether NRP-1 depletion could inhibit the growth and metastasis of gastric cancer cells, and whether NRP-1 displays its role as co-receptors by interacting the VEGF, EGF and HGF-mediated pathways in gastric cancer cells.

## Methods

### Patients

A total of 141 consecutive patients with pathologically proved gastric cancer received curative resection at the Department of General Surgery, Qingdao Municipal Hospital in China, between January, 2011 and December, 2013. None of the patients received any preoperative anticancer treatments. Gastric cancer was staged according to the staging system by the American Joint Committee on Cancer [21]. The histologic classification of gastric carcinoma was performed according to the 2010 WHO histologic classification system and cell differentiation [21]. This study was approved by the Ethics Committee of Qingdao Municipal Hospital (Qingdao, China), and all patients gave their informed consent prior to the inclusion in the study.

### Cell culture, antibodies and reagents

Human gastric cancer cells MGC-803, SGC7901, BGC823, AGS, NCI-N87 and HGC-27 were obtained from the Type Culture Collection Cell Bank (Chinese Academy of Sciences Committee, Shanghai, China). Cells were cultured in RPMI-1640 medium supplemented with 10 % FBS (fetal bovine serum) at 37 °C in a humidified atmosphere of 5 % CO_2_. For antibodies (Abs) and reagents, please refer to Additional file 1.

### Immunohistochemistry of clinical specimens

Formalin-fixed specimens were transferred to 70 % ethanol, and subsequently paraffin-embedded, sectioned and mounted on 3-aminopropyltriethoxysilane-coated slides (Sigma, Shanghai, China). Antigen retrieval was performed by heating sections in a microwave in 0.01 M citrate buffer. Sections were blocked for 2 h, and incubated with a rabbit anti-human NRP-1 Ab (diluted 1:250) at 4 °C overnight. A standard horseradish peroxidase staining procedure was followed using a biotinylated secondary Ab (diluted 1:250, and immunoreactivity developed with Sigma FAST DAB (3,3’-diaminobenzidine tetrahydrochloride) and CoCl_2_ enhancer tablets. Sections were counterstained with hematoxylin. Normal rabbit sera were diluted 1:10 in PBS, and used for blocking and dilution of Abs. Negative controls were achieved by using irrelevant goat IgG at a dilution of 1:50. NRP-1 staining was assessed in 20 randomly selected fields per specimen using a semi-quantitative grading system, which reflected the proportion and intensity of staining present within the specimen. The staining intensity (Value A) was graded in a four-tier grading system: no staining (0), faint yellow (1), yellow (2) and brown (3). The extent of positive staining (Value B) was determined using a four-tier grading system based on the percentage of positive cells: ≤ 10 % (1), 11–40 % (2), 41–70 % (3), and ≥ 70 % (5). The immunohistological score for each specimen was calculated by A × B, and then each specimen was graded lower level (≤5) and higher level (>5).

### ShRNA expression vectors

The sequence targeting NRP-1 (GAGAGGUCCUGAAUGUUCC), corresponding to nucleotides 949–967 of human NRP-1 (GenBank NM_003873.5), was previously reported [13]. The chemically synthesized oligonucleotides were introduced into the pSuppressorNeo vector to generate NRP-1 shRNA as described previously [22, 23]. A scrambled shRNA vector (Sc-shRNA) targeting nonspecific sequence (UUCUCCGAACGUGUCACGU) served as controls.

### Establishment of stable transfectants, Cell viability analysis, Bromodeoxyuridine (BrdU) incorporation assay, Assessment of cell cycle, Transfection of siRNA, Quantitative reverse-transcription polymerase chain reaction (qRT-PCR), Migration assay, Immunoblotting, In situ Ki-67 proliferation index and Assessment of tumor vascularity

Detailed methods have been described previously [22, 23] and can be found in Additional file 1.

### Animal experiments

Six to 8-week-old male nude BALB/c mice (H-2b) were obtained from the Animal Research Center, The First Affiliated Hospital of Harbin Medical University, China. The experimental protocol was described previously [22, 23]. This study was approved (permit SYXK20020009) by the Animal Ethics Committee of Harbin Medical University (Harbin, China).

#### Tumorigenicity study

Cells (2 × 10^6^) were injected subcutaneously into groups of 10 mice. The animals were monitored every 3 days. Mice were sacrificed 24 days later, and tumors were harvested and weighed.

#### In vivo metastasis study

Cells (1 × 10^6^) were injected into groups of 8 mice via the tail vein. The mice were sacrificed 7 weeks later. Lungs were harvested, weighed, then sectioned and stained by hematoxylin/eosin.

#### Therapeutic effect study

Cells (5 × 10^6^) were injected subcutaneously into the flanks of mice. When tumors reached ~100 mm^3^, the mice were assigned to 2 groups of 8 mice, which received an intratumoral injection of Sc-shRNA or NRP-1 shRNA, respectively. The shRNA transfection solution was prepared by mixing shRNA vector, Lipofectamine2000 and serum-free medium. Each tumor received an injection of 50 μl shRNA transfection solution containing 200 μg shRNA. Two mice from each group were sacrificed 4 days after intratumoral injection for detecting the expression of NRP-1. Tumors were measured and mice killed 20 days later.

### Statistical analysis

Association between NRP-1 expression and clinicopathologic parameters was analyzed by Mann Whitey test. Other data are expressed as mean values ± standard deviation. Comparisons were made with a one-way analysis of variance (ANOVA) followed by a Dunnet’s test. *P* < 0.05 was considered statistically significant.

## Results

### NRP-1 expression in gastric cancer tissues and its correlation with clinicopathologic parameters

**Fig. 1:**
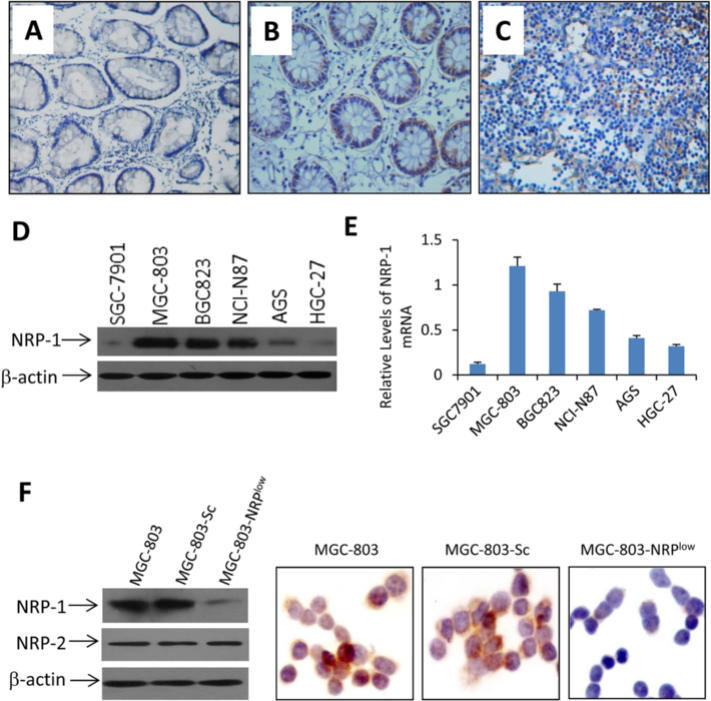
Expression of NRP-1 in gastric cancer tissues and cells. **a-c** Representative images of section from gastric normal mucosa (**a**), adenocarcinoma (**b**) and undifferentiated cancer (**c**), immunostained with an anti-NRP-1 antibody. **d** Lysates of human gastric cancer cells were immunoblotted. **e** The expression of NRP-1 mRNA was analyzed by qRT-PCR, and normalized to β-actin. **f** The expression of NRP-1 and NRP-2 was examined in lysates of parental MGC-803, MGC-803-Sc and MGC-803-SUR^low^ cells by immunoblotting. NRP-1 expression in the three cell lines was further detected by immunohistochemistry

Normal gastric mucosa had weak NRP-1 expression (Fig. 1a), whereas gastric cancer tissues expressed different levels of NRP-1 protein (Table 1). Representatively, gastric adenocarcinoma (Fig. 1b) expressed relatively lower levels, while undifferentiated gastric cancer (Fig. 1c), relatively higher levels of NRP-1. The level of NRP-1 expression was significantly correlated with clinical staging, tumor differentiation and pathological types of gastric cancer, but not with patients’ gender and age (Table 1).

**Table 1:** Relationship between NRP-1 protein expression and clinical pathological parameters

	Number	Lower level of NRP-1	Higher level of NRP-1	*P* value
Gender				0.738
Male	87	47	40	
Female	54	25	29	
Age median (range):53.4 (27–82)				0.345
<60	93	43	50	
≥60	48	29	19	
TNM stage				0.019
Stage 0	5	4	1	
Stage I	26	20	6	
Stage II	38	23	15	
Stage III	49	18	31	
Stage IV	23	7	16	
WHO histologic classification				0.046
Tubular	67	38	29	
Papillary	42	22	20	
Mucinous	13	4	9	
Poorly cohesive	11	3	8	
Uncommon histologic variants	8	5	3	
Differentiation				0.025
Well	35	27	8	
Moderate	74	36	38	
Poor	32	9	23	

Notes: NRP-1, Neuropilin-1. P value was estimated by Mann Whitney test

### NRP-1 expression in gastric cancer cell lines

Human gastric cancer cells expressed different levels of NRP-1 protein (Fig. 1d), and mRNA (Fig. 1e). MGC-803 cells expressing the highest level of NRP-1 were selected for generating stable transfectants. MGC-803 cells stably transfected with NRP-1 shRNA or Sc-shRNA were termed MGC-803-NRP^low^ and MGC-803-Sc, respectively (Fig. 1f). Compared with parental MGC-803 cells, MGC-803-NRP^low^ cells expressed significantly lower, while MGC-803-Sc cells expressed similar, levels of NRP-1. In addition, the three cell lines expressed similar levels of NRP-2 (Fig. 1f).

### NRP-1 depletion inhibits cell growth

**Fig. 2:**
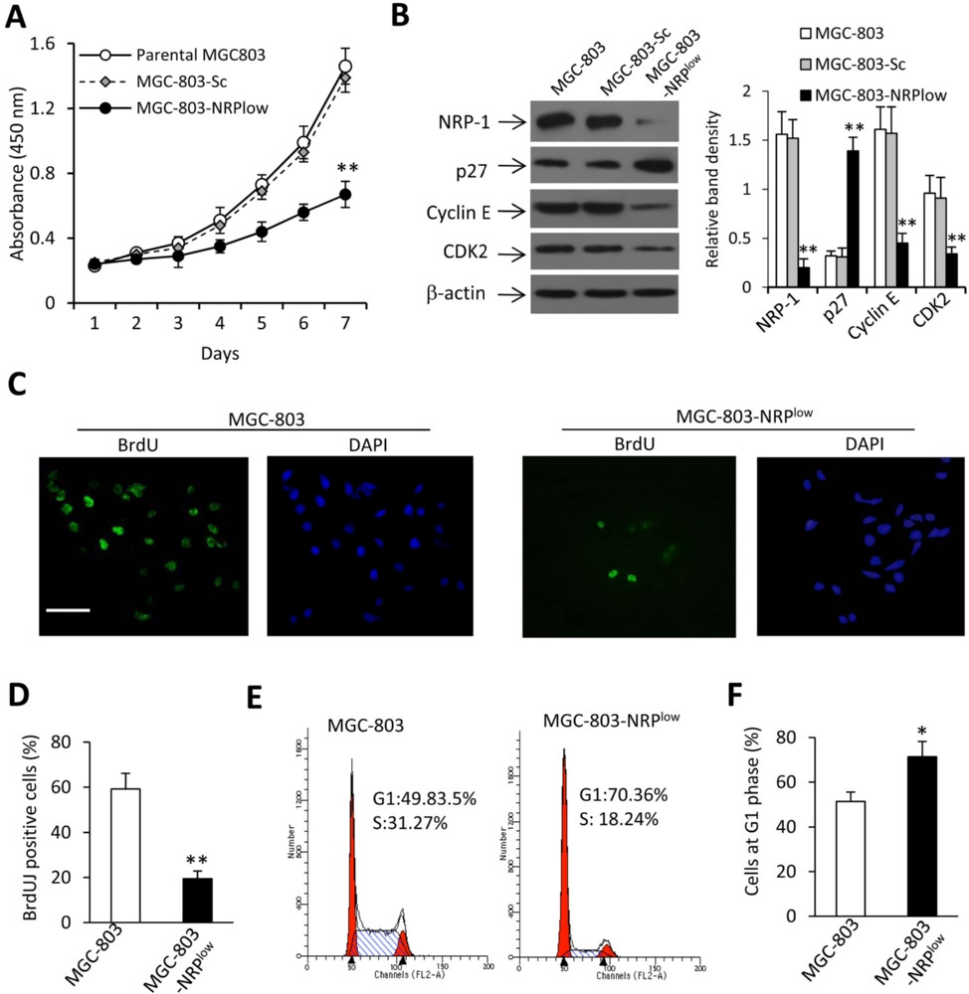
Cell proliferation *in vitro*. **a** MGC-803, MGC-803-Sc and MGC-803-NRP^low^ cells were cultured for 7 days, and their viability measured. **b** Cellular expression of NRP-1, p27, cyclin E and CDK2 proteins was detected by immunoblotting. **c**-**f** MGC-803 and MGC-803-NRP^low^ cells were cultured for 4 days. **c**, **d** Cell proliferation was analyzed by a BrdU assay. **c** The percentage of BrdU-positive cells was plotted. **d** Representative images from the nuclei of proliferating cells stained green by BrdU, and the nuclei of all cells stained blue by DAPI. Magnification bar = 200 μm. **e** Cells were subjected to flow cytometry to measure cell cycle distribution. **f** The percentages of cells at the G1 phase were plotted. The density of each band was measured and normalized to β-actin. “*” (*P* < 0.05) and “**” (*P* < 0.001) indicate a significant difference from MGC-803 cells

MGC-803-NRP^low^ cells had significantly lower viability, while MGC-803-Sc cells had the similar viability, compared with parental MGC-803 cells (Fig. 2a). We examined the expression of cyclin E, CDK2 and p27, which play key roles in cell proliferation and cycle progression [24]. The results showed that MGC-803-NRP^low^ cells expressed significantly lower levels of cyclin E and CDK2, and a higher level of p27, than parental MGC-803 cells (Fig. 2b). However, MGC-803-Sc cells expressed similar levels of the above proteins compared with parental MGC-803 cells (Fig. 2b). We then examined cell proliferation by means of BrdU-DAPI staining (Fig. 2c, d). MGC-803-NRP^low^ cells had a significantly lower rate of proliferation than parental MGC-803 cells. Specifically, 58.5 ± 7.3 % of parental MGC-803 cells, while only 19.6 ± 3.8 % of MGC-803-NRP^low^, were BrdU-positive (Fig. 2c, d). We further detected cell cycle distribution by flow cytometry. The fraction of cells at the G1 phase was significantly higher in MGC-803-NRP^low^ cells compared with parental MGC-803 cells (Fig. 2e, f).

### NRP-1 depletion inhibits cell migration

**Fig. 3:**
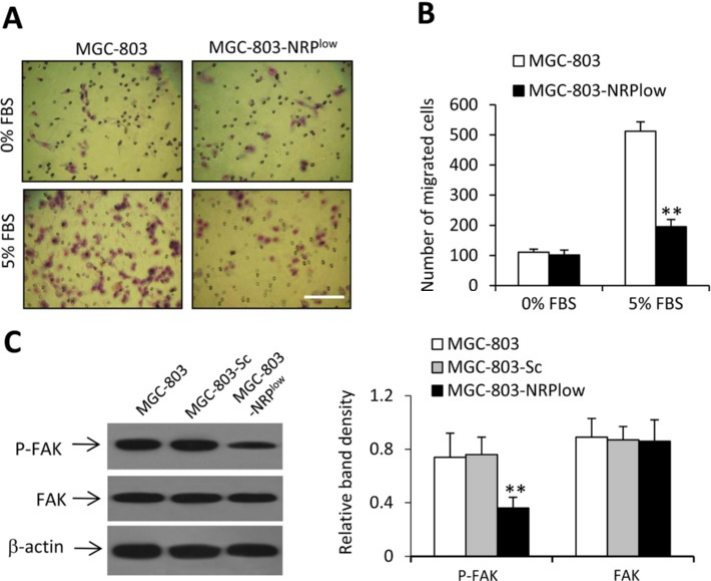
Cell migration. **a, b** MGC-803 and MGC-803-NRP^low^ cells were subjected to migration assays, in the presence or absence of 5 % FBS as chemoattractants. **a** Migrated cells were visualized using Giemsa staining. Magnification bar = 200 μm. **b** Numbers of migrating cells were counted. **c** MGC-803, MGC-803-Sc and MGC-803-NRP^low^ cells were immunoblotted to determine the expression of FAK and P-FAK. The density of each band was measured and normalized to β-actin. “**” (*P* < 0.001) indicates a significant difference from parental MGC-803 cells

The number of migrated MGC-803-NRP^low^ cells was significantly lower than that of parental MGC-803 cells (Fig. 3a, b). It has been reported that NRP-1 interacts the VEGF/VEGFR2 pathway, resulting in phosphorylation of FAK, a key factor for cell migration [11]. Here we showed that MGC-803-NRP^low^ cells expressed lower, but MGC-803-Sc cells expressed similar, levels of P-FAK, compared with parental MGC-803 cells (Fig. 3c). The three cell lines expressed similar levels of FAK (Fig. 3c).

### NRP-1 depletion inhibits tumorigenesis, tumor growth and metastasis

**Fig. 4:**
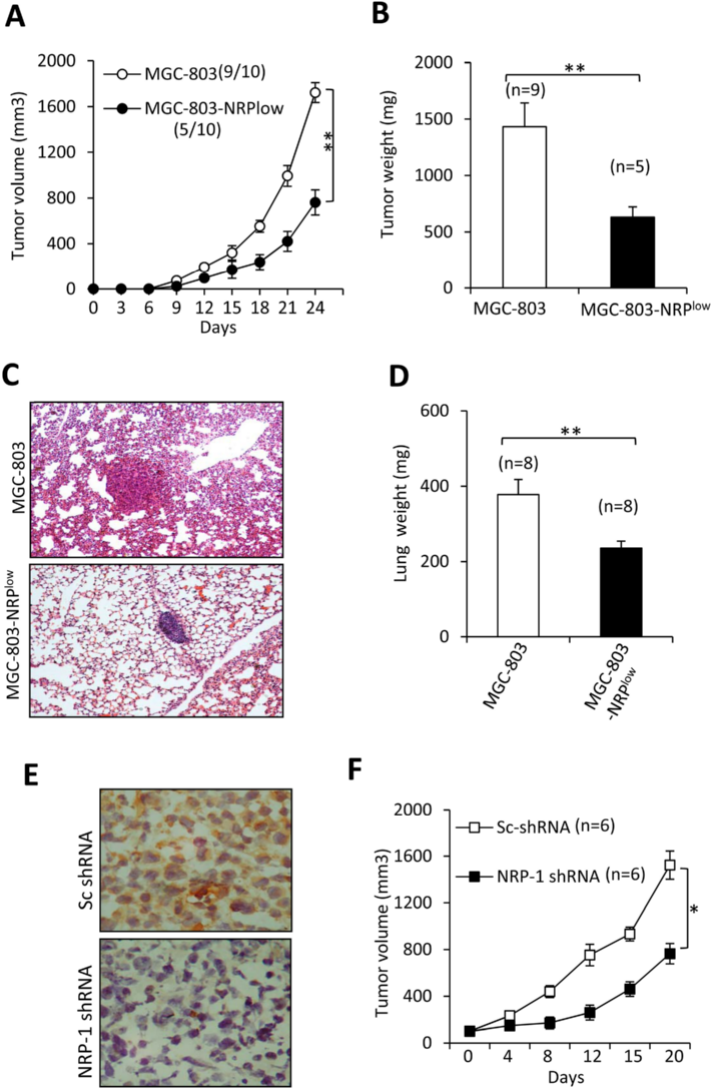
Tumorigenesis, metastasis and tumor growth *in vivo*. **a**, **b** MGC-803 and MGC-803-NRP^low^ cells were subcutaneously inoculated into the mice. **a** Tumor volumes were recorded. The number of tumor-bearing mice/ total number of mice are shown in brackets. **b** Tumors were weighed at the end of experiments. **c**, **d** The above cells were intravenously injected into the mice. The mice were killed 7 weeks later to harvest the lungs, which were sectioned and stained by hematoxylin/eosin (**c**), and weighed (**d**). **e**, **f** BGC823 tumors were established in mice, and injected with control Sc-shRNA or NRP-1 shRNA when they reached ~100 mm^3^ in volume. **e** Illustrated are representative tumor sections prepared 4 days following intratumoral injection. NRP-1 was immunostained brown by an anti-NRP-1 Ab. Magnification × 400. **f** The sizes of tumors were measured. “n” indicates the number of tumors. “*” indicates *P* < 0.05, and “**”, *P* < 0.001

Based on the in vitro results, we further investigated the effects of NRP-1 in animal models. Since MGC-803-Sc cells did not show significant different from parental cells in expression of the above proteins involved in cell proliferation and migration, we only used parental MGC-803 cells as controls in the following experiments. As shown in Fig. 4a, subcutaneous tumors were detected in 9 out of 10 mice injected with parental MGC-803 cells, but in only 5 out of 10 mice receiving MGC-803-NRP^low^ cells. In addition, MGC-803-NRP^low^ tumors grew to 761.8 ± 104.3 mm^3^ (1432.1 ± 209.6 mg in weight), whereas control MGC-803 tumors reached 1722.4 ± 89.6 mm^3^ (633.1 ± 90.7 mg in weight), 24 days following cell inoculation (Fig. 4a, b). The mice injected with MGC-803-NRP^low^ cells had fewer and smaller metastatic nodules in their lungs (with an average weight of 235.7 ± 18.5 mg), compared with those injected with parental cells (with an average weight 378.1 ± 40.3 mg) (Fig. 4c, d). In the study for testing the therapeutic effects, we established subcutaneous tumors by using another gastric cancer cell line, BGC823, which was also shown to express higher levels of NRP-1 (Fig. 1d, e). When tumors reached ~ 100 mm^3^, they were injected with either Sc-shRNA or NRP-1 shRNA. As shown in Fig. 4e, intratumoral delivery of NRP-1 shRNA resulted in downregulation of NRP-1 *in situ*, compared with Sc-shRNA. BGC823 tumors treated with NRP-1 shRNA were significantly smaller (769.1 ± 82.4 mm^3^) than those treated with Sc-shRNA (1521.7 ± 118.5 mm^3^), 20 days after treatment started (Fig. 4f).

### NRP-1 depletion inhibits cell proliferation in situ and tumor angiogenesis

**Fig. 5:**
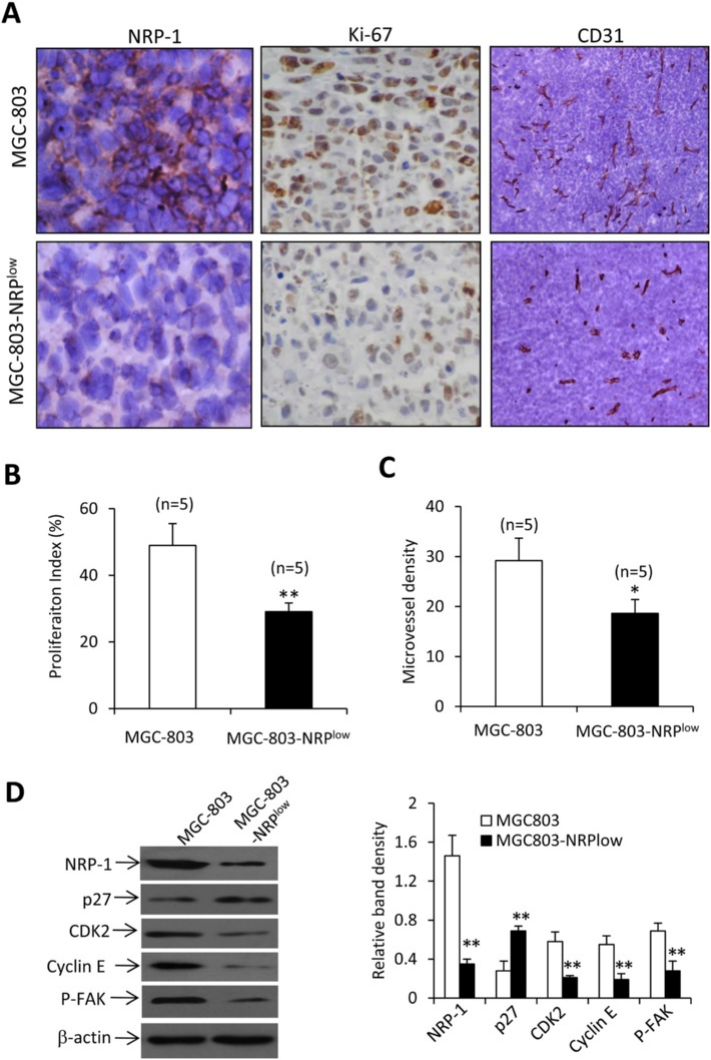
Tumoral angiogenesis and cell proliferation *in situ*. **a** Illustrated are representative sections prepared from tumors in Fig. 4b. Sections were stained with antibodies against NRP-1 (left panel), Ki67 (middle panel) or CD31 (right panel). The cell proliferation index (**b**) and microvessel density (**c**) were quantified. “n” indicates the number of tumors examined. **d** Homogenates prepared from tumors in Fig. 4b were immunoblotted. The density of each band was measured and normalized to that of β-actin. “*” (*P* < 0.05) and “**” (*P* < 0.001) indicate a significant difference from MGC-803 tumors

The tumors harvested in Fig. 4b were immunostained for examining cell proliferation and tumor angiogenesis. In consistence with the *in vitro* results (Fig. 1f), MGC-803-NRP^low^ tumors expressed markedly lower levels of NRP-1 proteins than MGC-803 tumors (Fig. 5a). NRP-1 depletion significantly inhibited cell proliferation and tumoral angiogenesis (Fig. 5a-c). The expression of key molecules *in vivo* (Fig. 5d) was consistent with that obtained *in vitro*.

### NRP-1 depletion inhibits VEGF-, EGF- and HGF-activated pathways

**Fig. 6:**
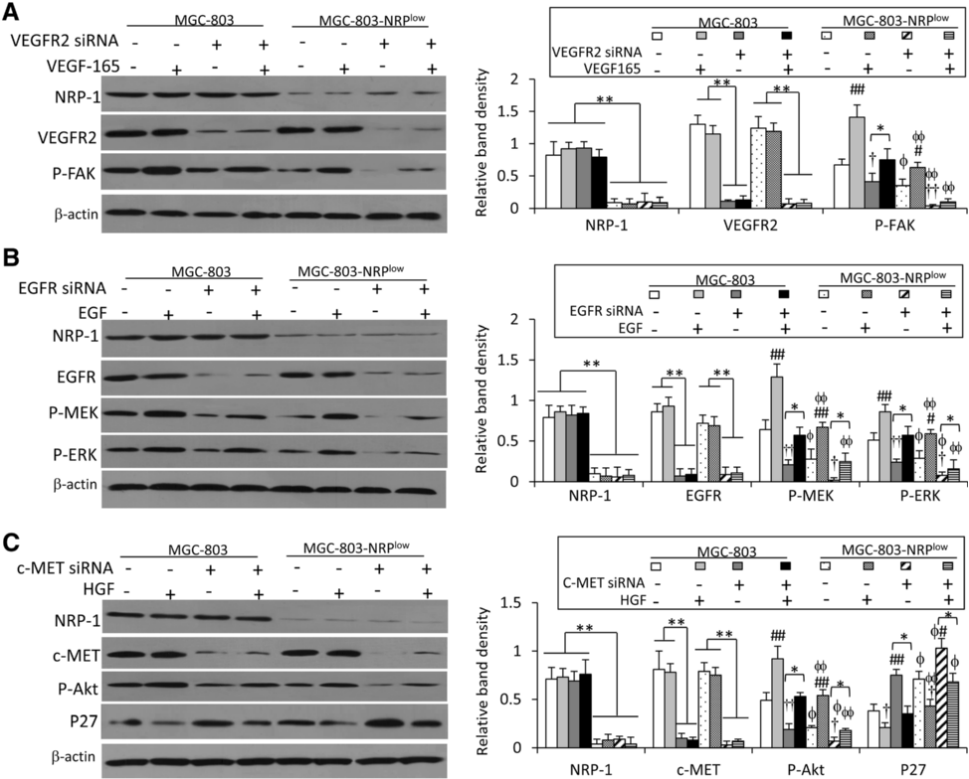
NRP-1 depletion influences the activation of VEGF/VEGFR2, EGF/EGFR and HGF/c-MET pathways. MGC-803 and MGC-803-NRP^low^ cells were transfected with siRNAs targeting VEGFR2 (**a**), EGFR (**b**) or c-MET (**c**). The cells were then cultured for 24 h in the presence or absence 100 ng/ml of recombinant VEGF-165 (**a**), of 10 ng/ml of recombinant EGF (**b**) or 100 ng/ml of recombinant HGF (**c**) proteins, respectively. Cell lysates were immunoblotted to determine the expression of key proteins involved in the above pathways as indicated. The density of each band was measured and normalized to β-actin. “*” indicates *P* < 0.05, and “**”, *P* < 0.001. “#” (*P* < 0.05) and “##” (*P* < 0.001) indicate a significant increase, and “†” (*P* < 0.05) and “††” (*P* < 0.001), a significant reduction, from untreated respective cells. “ϕ” (*P* < 0.05) and “ϕϕ” (*P* < 0.001) indicate a significant difference from MGC-803 cells with the same treatments

NRP-1 is shown to participate as co-receptors [10–14] in the activation of the VEGF/VEGFR2, EGF/EGFR and HGF/c-Met pathways, which play key roles in the proliferation and metastasis of gastric cancer cells [17, 18, 25]. Therefore, we investigated whether depletion of NRP-1 could suppress the activation of these pathways stimulated by respective ligands in gastric cancer cells. MGC-803 and MGC-803-NRP^low^ cells were transfected with siRNAs targeting VEGFR2, EGFR and c-Met, and then incubated with recombinant human VEGF-165, EGF and HGF proteins, respectively. Transfection of siRNAs targeting VEGFR2, EGFR and c-Met resulted in markedly downregulation of VEGFR2, EGFR and c-Met, but had no effect on the expression of NRP-1, in both MGC-803 and MGC-803-NRP^low^ cells (Fig. 6a-c).

Incubation of recombinant VEGF-165 protein had no effect on the expression of NRP-1 or VEGFR2, but markedly increased the levels of P-FAK, a downstream factor of VEGFR2 pathways [11], in MGC-803 and MGC-803-NRP^low^ cells (Fig. 6a). Transfection of VEGFR2 siRNA significantly reduced the level of P-FAK in MGC-803 and MGC-803-NRP^low^ cells (Fig. 6a). The level of P-FAK was significantly lower in MGC-803-NRP^low^ cells than MGC-803 cells, when they received the same treatments, either untreated, incubation with VEGF-165 protein, transfection with VEGFR2 siRNA, or the combination (Fig. 6a).

NRP-1 regulates the EGFR signaling pathways, resulting in activation of downstream signaling cascades including the MEK/ERK pathway [13]. Here we showed that incubation of recombinant EGF protein had no effect on the expression of NRP-1 or EGFR, but increased the levels of P-MEK and P-ERK proteins in parental MGC-803 and MGC-803-NRP^low^ cells (Fig. 6b). EGFR siRNA transfection significantly inhibited the expression of P-MEK and P-ERK in parental MGC-803 cells and MGC-803-NRP^low^ cells (Fig. 6b). The levels of P-MEK and P-ERK were significantly lower in MGC-803-NRP^low^ cells than MGC-803 cells, when they received the same treatments, either untreated, incubation with EGF protein, transfection with EGFR siRNA, or the combination (Fig. 6b).

The HGF/c-Met pathway is involved in the progression of gastric cancer by activating Akt pathways [18]. Here we demonstrated that incubation of recombinant HGF protein had no effect on the expression of NRP-1 or c-Met, but increased the levels of P-Akt and reduced the levels of p27 in parental MGC-803 and MGC-803-NRP^low^ cells (Fig. 6c). Transfection of c-Met siRNA significantly downregulated the expression of P-Akt but increased the expression of p27 in parental MGC-803 cells and MGC-803-NRP^low^ cells (Fig. 6c). The levels of P-Akt were significantly lower, while the levels of p27 were significantly higher, in MGC-803-NRP^low^ cells than MGC-803 cells, when they received the same treatments, either untreated, incubation with HGF protein, transfection with c-Met siRNA, or the combination (Fig. 6c).

## Discussion

NRP-1 has been shown to participate in the proliferation of cells from hepatocellular carcinoma [2], lung cancer [6], leukemia and lymphoma [8] and medulloblastoma [9]. In accord, the present results have demonstrated that depletion of NRP-1 inhibited the proliferation of gastric cancer cells by inducing cell cycle arrest in the G1/S phase through upregulating p27 and downregulating cyclin E and CDK2. P27 is implicated in the negative regulation of cell cycle progression from G_1_ to S phase by binding to and modulating the activity of CDKs [24]. Conversely, the cyclin E/CDK2 complex promotes progression from G_1_ to S phase by triggering the initiation of DNA replication [24].

Distant metastasis is the leading cause of mortality for gastric cancer, and is a major treatment obstacle [21]. Anti-angiogenic therapy, such as VEGF inhibition, continues to be a focus for developing anticancer drugs for gastric cancer [16]. However, VEGF inhibition demonstrates only transient and invariable benefits, and can also induce hypertension and renal toxicity due to its off-target effects by blocking the cellular signaling of VEGFR1 [26]. The recognition that NRP-1 is engaged by specific VEGF isoforms has expanded the landscape for developing modulators of VEGF-dependent signaling [27]. Here we showed that NRP-1 depletion inhibited VEGF-activated VEGF/VEGFR2 pathway, which is crucial for tumor angiogenesis by regulating the phosphorylation of FAK [28], a key factor in cell migration and metastasis [29]. In support of our results, blockage of NRP-1 inhibited tumor growth and VEGF-mediated angiogenesis [11]. The VEGF/NRP-1 pathway is also involved in cancer cell proliferation by activating Akt, leading to sequential p27 downregulation [30].

NRP-1 interacts with EGFR and promotes its signaling cascade elicited upon EGF stimulation in cancer cells [13]. The EGF/EGFR pathway is involved in the progression of gastric cancer [17]. In accord, the present study has demonstrated that NRP-1 silencing counteracted ligand-induced EGFR activation, evidenced by the reduced phosphorylation of MEK and ERK, two downstream factors of the EGFR pathway [31]. The MEK/ERK pathway participates in the progression of gastric cancer by contributing to cell proliferation and metastasis [17, 32].

NRP-1 is also implicated in the activation of HGF-induced signaling and cellular responses in cancer cells [14]. The involvement of HGF/c-Met pathway leads to the activation of PI3K/Akt signaling pathway in gastric cancer cells [18]. Accordingly, we have demonstrated herein that NRP-1 depletion attenuated HGF-stimulated the activation of HGF signaling pathway, evidenced by reduced activation of Akt.

**Fig. 7:**
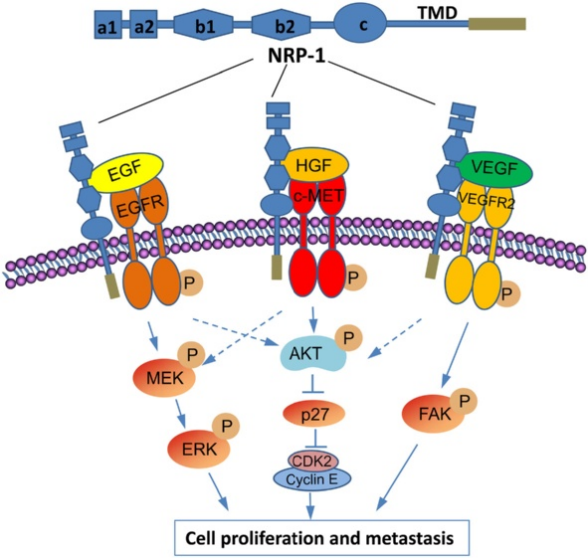
NRP-1 structure and a proposed model of interactions of NRP-1 as multifunctional co-receptors with VEGF/VEGFR2, EGF/EGFR and HGF/c-MET pathways in gastric cancer cells. NRP-1 molecule consists of five extracellular domains, a single-pass transmembrane domain (TMD) and a short cytosolic tail. “→” indicates promotion, positive regulation or activation; “⊥”, inhibition, negative regulation or blockade. A dotted line indicates uncertain mechanisms, while a solid line, identified mechanisms. Abbreviations: CDK, cyclin-dependent kinase; EGF, epithelial growth factor; EGFR, epithelial growth factor receptor; ERK, extracellular-signal-regulated kinase; FAK, focal adhesion kinase; HGF, hepatocyte growth factor; MEK, mitogen-activated protein kinase; NRP-1, neuropilin-1; VEGF, vascular endothelial growth factor; VEGFR, vascular endothelial growth factor receptor

The general domain structure of NRP-1 [1] and proposed mechanisms for its role as co-receptors in the proliferation and metastasis of gastric cancer cells are summarized in Fig. 7. NRP-1 co-interacts with VEGF/VEGFR2 [10–12], EGF/EGFR [13] and HGF/c-Met [14] pathways. The activation of the VEGFR2 pathway results in the phosphorylation of FAK, which promotes cell metastasis [11, 28]. The activation of the c-Met pathway induces sequential activation of Akt [25], which in turn leads to downregulation of p27 [24, 33]. P27 negatively regulates cell cycle progression by modulating the activity of CDK2/cyclin E complex [24]. The activation of EGFR pathway leads to the phosphorylation of MEK and ERK, which contributes to the proliferation and metastasis of gastric cancer cells [17, 32]. Although not investigated in the present study, the cross-talks among the above signaling pathways may also be involved in cancer cell proliferation and metastasis [31].

## Conclusions

In summary, NRP-1 is overexpressed in gastric cancer tissues, and its expression correlates with the clinical staging, tumor differentiation and pathological types of gastric cancer. NRP-1 depletion inhibited the proliferation and migration of gastric cancer cells, and suppressed their ability to form tumors and to metastasize to lungs, and therapeutic NRP-1 shRNA inhibited the growth of established gastric tumors in experimental animals. NRP-1 depletion counteracted the activation of VEGF/VEGFR2, EGF/EGFR and HGF/c-Met pathways stimulated by respective ligands, indicating that NRP-1 acts as multifunctional co-receptors in gastric cancer cells. Taken together, the present results suggest that NRP-1 may be a valuable biomarker and potential therapeutic target for gastric cancer, particularly for those expressing higher levels of NRP-1.

## Additional file

Additional file 1: **Supplementary materials.** (DOCX 23 kb)

## Abbreviations

ANOVA: Analysis of variance
BrdU: Bromodeoxyuridine
CDK: Cyclin-dependent kinase
EGF: Epithelial growth factor
EGFR: Epithelial growth factor receptor
ERK: Extracellular-signal-regulated kinase
FAK: Focal adhesion kinase
FBS: Fetal bovine serum
HGF: Hepatocyte growth factor
MEK: Mitogen-activated protein kinase
NRP-1: Neuropilin-1
qRT-PCR: Quantitative reverse-transcription polymerase chain reaction
VEGF: Vascular endothelial growth factor
VEGFR: Vascular endothelial growth factor receptor
