# Supplementary material for: Neuropilin-1 is associated with clinicopathology of gastric cancer and contributes to cell proliferation and migration as multifunctional co-receptors
Source: J Exp Clin Cancer Res. 2016 Jan 22;35:16. doi: 10.1186/s13046-016-0291-5 (PMC4722781; doi:10.1186/s13046-016-0291-5)
Supplement: Supplementary file 1 — Supplementary materials. (DOCX 23 kb) [file 13046_2016_291_MOESM1_ESM.docx]

**Additional Detailed Methods**

**Antibodies and reagents**

The antibodies (Abs) used in this study included Abs against human NRP-1, NRP-2, p27, cyclin-dependent kinase 2 (CDK2), cyclin E, FAK (focal adhesion kinase), pFAK^Tyr576/577^ (phosphorylated FAK), p27 and β-actin (Santa Cruz Biotechnology, Santa Cruz, CA, USA); Abs against AKT, phosphorylated AKT (p-AKT, Ser^473^), Phosphorylated MEK1/2 (P-MEK, Ser217/221), ERK (extracellular signal-regulated kinase) 1/2, and phosphorylated ERK1/2 (p-ERK, Thr^202^/Thyr^204^) (Cell Signaling Technology, Danvers, MA, USA), an Ab against CD31 (Thermo Scientific, Beijing, China), and an anti-Ki67 Ab (Abcam, Cambridge, MA, USA). Recombinant human VEGF-165, EGF and HGF proteins were purchased from Thermo Scientific, Beijing, China. DAPI (4',6-diamidino-2-phenylindole) was purchased from Sigma.

**Establishment of stable transfectants**

Cells were seeded in 10-cm plastic dishes and grown to 67% confluence at which point they were transfected with 4 μg of each vector using Lipofectamine2000 (Invitrogen). They were detached with trypsin after transfection for 48 h, and seeded in selection medium containing geneticin (G418) (500 μg/ml). Stable transfectants were selected at 4 weeks of culture.

**Cell viability assay**

The Cell Counting Kit-8 (CCK-8) (Dojindo Molecular Technologies, Inc. Beijing, China) was used to determine cell viability. Cells were seeded at 1 × 10^3^ cells /well in 96-well plates. At different time points, the culture medium was replaced with 100 μl of fresh medium containing 10 μl of CCK-8 solution. The cells were further incubated for 2 h at 37°C, and the optical density (OD) at 450 nm was measured. The experiment was repeated thrice.

**Bromodeoxyuridine (BrdU) incorporation assay**

Cell proliferation was measured using the 5-bromo-2-deoxyuracil (BrdU) labeling kit (Boster Biological Technology Ltd., China). Briefly, BrdU was added to the culture medium at the final concentration of 10 μM. BrdU-labeled indices were measured by visually scoring nuclei stained with 4′, 6-diamidino-2-phenylindole (DAPI) in 50 to 100 cells in 20 independent visual fields. Thereafter, BrdU-positive cells were scored as a percentage of the total cell number. The experiment was repeated thrice.

**Gene knockdown by siRNAs**

Small interfering RNA transfections were carried out as recommended by the manufacturer (Santa Cruz Biotechnology, Inc). Cells were plated (200 000 cells per well, six-well plates) in 10% serum overnight and transfected with 60 pmol control siRNA (an irrelevant control sequence, sc-37007) or VEGFR2 siRNA (sc-29319), EGFR siRNA (sc-29301) or c-Met siRNA (sc-29397) for 6 h. Cells were allowed to grow for 48 h and lysates were analyzed.

**Assessment of cell cycle**

Cells were seeded at 5.0×10^5^ cells/well in 6-well plates, cultured for 48 h, and then harvested. The percentage of cells at G1 and S phases was determined with a cell cycle detection kit (BD Biosciences, Beijing, China) using flow cytometry with a Beckman Coulter Epics Altra II cytometer (Beckman Coulter, California, USA). The experiments were repeated thrice.

**Migration assay**

Transwells (8 μm pore size) and the basement membrane matrix Matrigel were purchased from BD Bioscience (San Jose, CA, USA). Cells (2×10^4^) suspended in 200 μl of serum-free medium were seeded on the polycarbonate membrane in a transwell culture chamber, and the lower chamber was filled with 750 μl of medium with 5% FBS as chemoattractants. After incubation for 12 h at 37°C in a humidiﬁed atmosphere of 5% CO_2_, the transwell culture chamber was washed with PBS and the cells on the top surface of the polycarbonate membrane were removed. Cells that migrated to the bottom surface of the insert were fixed with methanol and stained by Giemsa stain. The cells were counted based on digital images of 5 fields taken randomly at × 200. The experiments were repeated thrice.

**Quantitative Reverse-Transcription Polymerase Chain Reaction (qRT-PCR)**

Briefly, total RNA was extracted from the cells, and cDNA was synthesized. The reaction mixtures for qRT-PCR were prepared with the primers for NRP-1 (Forward: 5'-GGAGCTACTGGGCTGTGAAG-3'; and Reverse: 5'-ACCGTATGTCGGGAACT

CTG-3') and β-actin (Forward: 5’- AGCGAGCATCCCCCAAAGTT -3’; Reverse: 5’- GGGCACGAAGGCTCATCATT-3’), and analyzed by MX3000P Real-time PCR systems (Stratagen, USA). Experiments were performed as triplicate, and the data were calculated by ∆∆Ct methods.

**Immunoblotting**

Protein concentrations of cell or tissue lysates were determined using the Bio-Rad protein assay (Bio-Rad, Richmond, CA, USA). Lysates were resolved on SDS-polyacrylamide gels, and the proteins transferred to PVDF membranes, and immunoblotted as previously described [23-24]. The density of each band was measured using the FR200 densitometric analysis program (Shanghai, China). In preliminary experiments, serial dilutions of lysates containing 2.5, 5, 10, 20, 40 or 80 µg of protein were immunoblotted. Band intensities were measured and plotted against protein amounts to generate a standard curve, and the amount of protein for each immunoblot was determined.

**In situ Ki-67 proliferation index**

Formalin fixed tumor specimens were transferred to 70% ethanol, and subsequently paraffin-embedded and sectioned. Tumor sections were rinsed with PBS, blocked with 3% BSA for 2 h, and incubated with an anti-Ki-67 Ab at 4^o^C overnight. They were subsequently incubated for 30 min with the appropriate secondary Ab using the Ultra-Sensitive TMS-P kit (Zhongshan Co., Beijing, China), and immunoreactivity developed with Sigma FAST DAB (3,3’-diaminobenzidine tetrahydrochloride) and CoCl_2_ enhancer tablets (Sigma-Aldrich, Shanghai, China). Sections were counterstained with hematoxylin, mounted, and examined by microscopy. The Ki-67 positive cells were counted in 10 randomly selected × 400 high-power fields under microscopy. The Ki-67 proliferation index was calculated according to the following formula: the number of Ki-67 positive cells/ the total cell count × 100%.

**Assessment of tumor vascularity**

Briefly, 5 μm tumor sections were immunostained with an anti-CD31 Ab and examined under microscopy. Stained vessels were counted in ten blindly chosen random fields at 400 × magnification, and the microvessel density was recorded.
